# Teaching crisis management before and after the pandemic: Personal reflections

**DOI:** 10.1177/01447394221087889

**Published:** 2022-04-15

**Authors:** Paul ‘t Hart

**Affiliations:** 8125 Utrecht University, Utrecht, Netherlands

**Keywords:** Crisis leadership, crisis management education, transboundary crises, pandemic, covid 19, public management

## Abstract

This reflective contribution tells the story of a veteran public sector crisis management (CM) researcher’s 35-year journey with educating students and CM practitioners, It offers preliminary insights about how the pandemic experience might – and should – induce a significant rethink of how educators conceptualize the nature of crises and the challenges governments and public agencies face in coping with them.

## Humble beginnings

I first got involved in crisis management education for public servants in the mid-1980s. Having worked as a research assistant to my professor and future PhD-supervisor Uriel Rosenthal, I had trawled through the then scant literature – the functioning of governments and leaders in situations of threat, urgency and uncertainty had been studied extensively by international relations (IR) scholars (Hermann, 1972; George and Smoke, 1974; Jervis, 1976; Brecher, 1979; Lebow, 1981) but there was next to no systematic empirical research on how public policymakers and agencies coped with domestic emergencies, such as economic shocks, natural and major industrial or infrastructural disasters, riots and terrorism.

Studying how governments prepare for, act during and move on from such extreme events was seen as a somewhat odd and marginal undertaking, particularly in prosperous and stable democratic polities such as the Netherlands and Sweden (where I soon started collaborating with like-minded colleagues). The main game in political science and the fledgling field of public administration had been elsewhere – democracy versus autocracy, the performance of different types of democracies, the evolution of party systems, the rise of ‘floating voters’, the growth and power of bureaucracy. Neo-institutionalism and new public management were just around the corner. Outside of IR, the concept of ‘crisis’ was mainly used by leftist scholars to highlight structural tensions in postwar liberal democracies and their welfare states.

In his pioneering work on crisis decision making in the Netherlands (Rosenthal, 1986) and the cross-jurisdictional comparative case study work, he, I and a small band of colleagues at Leiden University were undertaking at the time, we had to borrow concepts, propositions and methodologies from these IR scholars as well as from disaster sociology, safety science, business management, and psychological studies of humans and groups operating under stress (Rosenthal, 1986; Rosenthal et al., 1989, 1991; Rosenthal and Pijnenburg, 1991; Rosenthal and ’t Hart, 1991; t Hart et al., 1993).

But the times suited us. The 1973 OPEC oil shock had provided a first demonstration of what might happen when one of the foundations of the prevailing political-economic settlement – stable low prices of oil and natural gas – was removed overnight, with winter coming on. Governments and businesses were forced to improvise, take highly consequential decisions rapidly, under duress, and in an environment of high uncertainty. It proved a prelude to the contemporary ‘risk society’, in which we have become acutely aware of the unintended consequences of our reliance on the sophisticated but fallible socio-technical systems that now underpin our way of life.

### Momentum

Just as we were shaping up as a research team, the 1980s and 1990s provided a string of ‘rude surprises’ including the petrochemical catastrophe in Bhopal, India in 1984, Chernobyl, the Exxon Valdez oil spill in Alaska, the Challenger and Columbia space shuttle accidents, crowd disasters at rock concerts and soccer matches, waves of sectarian, ultra-leftist, and regionalist terrorism in West-Germany, Spain, northern Ireland as well as scores of urban riots in unlikely countries such as Denmark, the Netherlands and Switzerland. The UK experienced all of the above in what were grim decades.

These extreme events had to be ‘managed’, both in the here and now of emergency responses and the long grind of disaster recovery, as well as in the strategic domain of post-incident investigation, accountability, blame, and learning. Most governments were clearly not set up to do this. As a consequence, coping with crises went up a few notches in the order of their capacity-building priorities. Demand grew for evidence-based forms of crisis management expertise. All of a sudden, the fledgling research team we had built found itself riding a boom. Out team were now leaders in an emerging field – though still considered a niche interest in PA academia, CM had become a ‘hot’ area of applied research, training and consultancy (see, e.g., Stern and Sundelius, 2003).

### Toolkit and learning design

**Figure 1.**
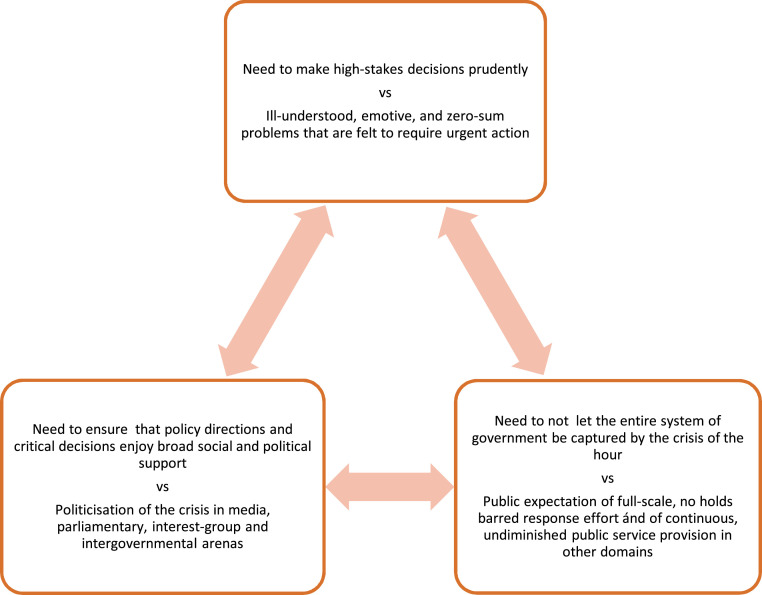
Crisis decision making as a ‘triple trilemma’ (source: author).

Our initial focus in both research and teaching had been on the dynamics of crisis decision making. Defining crises as perceived realities – a combination of high threat (i.e. problems that *must* be tackled in order to stave off chaos and loss), high uncertainty (i.e. ‘fog of war’ creating a problematic information environment) and high urgency (i.e. no time to play with in deciding upon a course of action and in deploying resources accordingly), we presented crisis decision-making as a dilemma-ridden balancing act (see Figure 1).

Drawing on the dozens of cases we had researched that showed common patterns of performing this balancing act regardless of the specific type of incident or the jurisdiction at play, we designed both historical and hypothetical role-playing exercises (‘serious gaming’) as the centrepiece of our educational offerings. Course participants ranging from third year undergraduates to mayors, police leaders, armed forces commanders, policy bureaucrats and later on entire executive boards and even national cabinets were put into decision-making, advisory and front-line delivery roles and fed information (and ‘noise’) about the evolving crisis, given opportunities to meet and make decisions or formulate recommendations for action, often under considerable time pressure. The scenarios were designed to let them experience the look and feel of real crisis events, to observe them at work, and to spend ample time debriefing what we had seen (Rosenthal and Pijnenburg, 1991; ‘t Hart, 1997). Scenario design, observations and debriefings were guided by the coping patterns we had observed in real cases. These included:

1. In the ***organization*** of decision making:
  o *Centralization* of decision making in (often informal) small elite groups
  o Prevalence of improvised modes of preparing and taking decisions
  o Strong reliance on (and therefore high potential power of) *expert advisers* (whose expertise is deemed essential to the crisis scenario at hand)
  o No-fuss *interagency collaboration* in some parts of the crisis response network, and high-fuss *bureaucratic politics* in others
2. In the ***information and communication*** processes feeding into and resulting from decision making
  o *Explosion in volume and speed* of upward, downward and outward communication flows
  o *Frantic search for useable information*, including by unconventional means when formal supply lines do not ‘deliver’
  o Increased likelihood of decision makers resorting to *historical analogies* in diagnosing situations and formulating responses
  o *In-group/out-group differentiation* in communication patterns: more effort is spent attending to trusted and liked parties, whereas communication with less-known, less-valued, less-trusted parties is eschewed
3. In **stress-induced coping behaviours**
  o *Constriction of time horizons*: strong focus on the here and now, crowding out attention for weeks, months and years ahead
  o *Threat-rigidity and escalation of commitment*: Increased likelihood of decision makers getting locked into their initial definition of the situation and/or initial policy decisions even in the face of overwhelming new and discrepant information
  o *Groupthink*: increased likelihood of premature and excessive concurrence-seeking among members of in-groups

In debriefings, we elaborated the observed incidence of these patterns and their observed effects on the quality of decision making, in terms of participants’ abilities to strike a situationally appropriate balance between the competing imperatives depicted in Figure 1 (see also Appendix 1). Over time we honed our craft of devising fit-for-learning-purpose scenarios (less is more – hence shorter, simpler, more dilemma-focused scenarios; images speak louder than words – hence the use of prerecorded news clips and other forms of audiovisual cueing). We learned to optimize debriefings (not an ornament but the core value-add – hence spending at least 50% of the time available on them instead of letting the role-playing rip).

### The 9/11 watershed

Then 9/11 happened. An outsized crisis that has a seismic geostrategic as well as a deep foreign and security policy impact within and far beyond the United States. At the same time, it affected the CM field deeply too. Not only did it inject bucketloads of money and top-level attention into CM research and education, it ‘securitised’ its agenda – all of a sudden (islamic) terrorism became the dominant risk and prism in contingency planning and capacity building, crowding out other salient risk factors and creeping crises such as climate change (cf. ASU News, 2021; Boin et al., 2021a).

Secondly, it highlighted what we had started to emphasize more and more in the years prior: that big crises cast long ‘shadows’, both forward and backward in time, and that the conventional focus on decision making, communication and coordination in the hot phase of the crisis provides too narrow a foundation for understanding CM capacity-building and professional development (‘t Hart et al., 1998; ‘t Hart and Boin, 2001; ‘t Hart and Sundelius, 2013).

Under much more intense media scrutiny and prone to protracted post mortem investigations, post 9/11-crises in a rapidly globalising world have become lengthier, multifaceted, politicised affairs, in which policy makers and agencies face with the twin challenges of managing messy adaptive processes of moving affected groups and communities on from shock, devastation and losses suffered, while simultaneous navigating the conflict-laden dynamics of investigation, accountability and learning that call into question what they did and did not do both prior to the crisis breaking and in the heat of the moment. Contemporary crises are at once cataclysmic and path-breaking. They open up windows of opportunity for terminating, reconstituting or creating public organizations, policies, laws and institutions (Boin et al., 2008; Helsloot et al., 2012).

### The pandemic experience

By the time Covid hit, the cottage industry that once was public sector CM research and education had turned into a globalised multidisciplinary behemoth, firmly embedded into the ‘security industrial complexes’ that sprung up in many Western nations. The pandemic both startled and rattled CM experts while drawing us into in feverish activity. I dual-tasked as a researcher presented with an absolutely unique ‘field lab’ of global proportions to observe crisis coping at work, and as a public scientist helping policymakers and mass public alike to make sense the bewildering turn of events as they unfolded in real time. I conducted dozens of webinars across the world, facilitated numerous ‘let’s-stop-and-think’ reflections sessions for senior executive teams, and participated in various ‘red-teaming’ scenario efforts designed to support strategic policymaking.

These pandemic-era experiences taught me five lessons that I am now resolved to integrate into my ongoing CM teaching, research and advisory work:

1. *The paradox of nation state authority and capacity*: the imposition of lockdowns, contact tracing, border closures and many other measures demonstrated the awesome authority that the state still holds over the bodies, privacy, freedom of movement and civil rights of citizens, while at the same time the ever-morphing virus transboundary threat eluded all but the most draconian forms of state intervention capacity to contain and eradicate it (see also Boin, 2019).
2. *The difficulty of ‘holding’ communities through pervasive and protracted uncertainty.* Wheareas initially publics accepted that crisis managers had to ‘take 100% of the decisions with only 50% of the requisite information’ as Dutch prime minister Rutte put it in April 2020, and experts – initially, predominantly medical experts – who could help plug the sensemaking gap were widely revered as ‘evidence-loving rockstars’, the rally around the flag did not last. Confronted with heavy constraints on their ways of life that were imposed seemingly on the basis of contestible guesstimates, the public wanted to know how long they would last and when they would end. When vaccines came on the scene right as the Northern hemisphere was grappling with second and third waves of the virus, the public wanted to have them instantaneously and be given clarity about the pace of the imminent ‘reopening of society.’ These pressures proved hard to resist, and consequently many policy makers fell into the trap of orchestrating dissatisfaction by prematurely providing roadmaps and other tokenistic forms of uncertainty reduction that time and again had to be adjusted, postponed or withdrawn altogether.
3. Despite the much richer information environments in which they operate compared their 20^th^ century predecessors, contemporary *crisis managers continue to be prone to presentism.* When today’s threat is comprehensive and bewildering, everybody’s focus is on it is here and now. The default perspective of even the most senior policymakers is reduced to the tactical - days and weeks – and nowhere near strategic. The pervasive demand from businesses and citizens for the provision ‘roadmaps’ out of the current confines of curfews and shutdowns and the loose talk in the media about ‘new normals’ and ‘post-Covid’ futures proved hard to resist. For most of 2020, the future was initially reduced to a race to produce and distribute vaccines, and when by early 2022 Omicron had finally shattered the hopes of vaccines ‘doing the trick’ of ridding the world of any further major virus-induced disruptions, many governments had done preciously little to consider the possibility of the current pandemic lasting as long as its global predecessors of ages past have done: over many years, in fits and starts. This same might well apply to many of the creeping crises the world now faces (Boin et al., 2021a).
4. In a world where everyone with a cell phone can ‘make news’ that goes viral, and where executive power is subjected to manifold formal and informal checks and balances, *achieving and sustaining narrative dominance* has become both a pivotal and a nearly impossible imperative for crisis managers, particularly in a protracted and all-pervasive crisis. More than ever before crises now unfold in framing contests over what the on the ground events should be taken to mean (Houlberg Salomonsen and ‘t Hart, 2020). After the honeymoon period of ‘experts-led’ crisis response heroism had worn off, the pandemic provided ample illustration of this fundamental characteristic of the modern crisis. Government truth claims and were being challenged more and more, as the language of ‘we’ gave way to ‘us and them’ (e.g. ‘the unvaccinated’). Popular support gave way to scepticism and – for some – frustration and rage, as restrictions continued being re-imposed and extended. In the framing contests between exogenous (the pandemic as a natural disaster) and endogenous (the pandemic as a man-made disaster) accounts of the course of the pandemic, the latter gained momentum, eating away at the legitimacy of public authorities (Boin et al. 2021b).
5. In the relatively scarce moments where the government bodies I had visibility of undertook more strategic policy planning exercises, I encountered entrenched *reluctance to consider worst case or even bad case scenarios.* The significance of this phenomenon – which has been well understood in terms of its cognitive, social-psychological and political drivers for a long time (Sunstein, 2009) – has become painfully clear during the pandemic. It beguiled policymakers time and again, leaving them on the back foot in the face of turns of events that should not have surprised them to the extent that they often did had they refrained from exasperation-driven wishful thinking and opened themselves up more to contemplating grimmer but entirely plausible scenarios.
6. Finally, the pandemic has done what I quickly learned from historians (e.g. Snowden, 2019) virtually every other pandemic in history has done, and what many of the creeping crises were are currently facing have begun to do: *acting as a pressure cooker in which pre-existing but hitherto ‘normalised’ social problems are aggravated, dramatised, and – not invariably but often enough – politicised.* So, it is not just that they have long shadows, crises also harbingers of societal conflict, choice and change.

### Teaching crisis management after the pandemic

If you agree with me that these lessons are pertinent and must find their way into how we design crisis education and professionalisation, then we have some way to go in reconsidering how we have tended to go about our business. Perhaps we need to start with *unlearning* a few of the assumptions that have underpinned our efforts for so long.

For one, we need to rid our mindsets, course content and simulation designs of the erroneous assumption that crises are short and sharp shocks to our systems, performance tests that governments can weather until they are over. We should *sensitize students and practitioners to the reality of protracted crises,* which means among others engaging them with the dynamics of crises that comprise multiple sequences of peak tension alternating with ‘in-between’ periods of quasi-normalcy stretching out over long periods of time, exasperation, fatigue, burnout, impatience, and high attrition rates permeating both society at large and the machinery of government itself.

Also, we need to let go of the idea that because the Hobbesian social contract places the burden of community expectations about restoration of order, security and safety upon the state and its functionaries, governments should ‘go at it alone’ in tackling crises. Many of the pivotal crises we currently face are transboundary in nature and their impacts exceed the governance capacity of the state. To tackle them as well as possible, business and community sectors’ problem-solving capacity needs to be tapped into, and transnational exchange and coordination will be called for. Consequently, we should place *strong emphasis on the need to develop capacity to manage crises collaboratively* – across agencies, levels of government, sectoral and national boundaries (Parker et al., 2020).

Thirdly, we will need to impress upon CM practitioners *the necessity of dodging myopia by cultivating capacity for maintaining a strategic outlook while navigating crises*. This entails maintaining dedicated, multidisciplinary analytical capacity to continuously engage in medium and long-term futuring. It also means giving these units license to engage decision makers in contemplating a broad spectrum of possibilities, including both worst-case scenarios and strategic opportunities.

Finally, we will need to shed the prescriptive 10-point plan style reputation management approach to crisis communication that has long permeated corporate crisis communication manuals and curriculums. It is built on assumptions of control and ‘image repair’ that are entirely unsuitable for the kinds of creeping and protracted crises governments are forced to contend with. Instead, we will need to confront CM practitioners with the *challenges of ‘performing authority’ in a crisis context of pervasive uncertainty, contestablemeaning making, and fired-up framing battles*.

The pandemic has forced not just confronted governments and public services with the limits of their capabilities for restoring order and control. It has also forced crisis management experts to rethink and adapt their craft. These reflections are just a hint of the scope and direction of the work that lies ahead of us.

## Appendix

Crisis decision making simulation observation form used in Leiden University Crisis Research Center education and training activities, 1988–1998 (Source: ‘t Hart, 1997: 209).
